# Identification of Immunomodulatory Signatures Induced by American Ginseng in Murine Immune Cells

**DOI:** 10.1155/2013/972814

**Published:** 2013-11-11

**Authors:** Jian Yan, Yonghui Ma, Fusheng Zhao, Weikuan Gu, Yan Jiao

**Affiliations:** ^1^Department of Primary/Public Health, Nursing College, Molecular Resource Center, University of Tennessee Health Science Center, Memphis, TN 38163, USA; ^2^Department of Orthopedics Surgery & Biomedical Engineering-Campbell Clinic, College of Medicine, University of Tennessee Health Science Center, Memphis, TN 38163, USA; ^3^Mudanjiang Medical University, Heilongjiang 157011, China

## Abstract

*Background*. American ginseng (*Panax quinquefolius*, AG) has been used for more than 300 years. Some of its claimed benefits can be attributed to the immunomodulatory activities, whose molecular mechanisms are largely unknown. *Methods*. Murine splenic cells from adult male C57BL/6 (B6) mice were isolated and divided into 4 groups to mimic 4 basic pathophysiological states: (1) normal naïve; (2) normal activated; (3) deficient naïve; (4) deficient activated. Then, different AG extracts were added to all groups for 24 h incubation. MTT proliferation assays were performed to evaluate the phenotypic features of cells. Finally, microarray assays were carried out to identify differentially expressed genes associated with AG exposure. Real-time PCR was performed to validate the expression of selected genes. *Results*. Microarray data showed that most of gene expression changes were identified in the deficient naïve group, suggesting that the pathophysiological state has major impacts on transcriptomic changes associated with AG exposure. Specifically, this study revealed downregulation of interferon-**γ** signaling pathway in the deficient group of cells. *Conclusion*. Our study demonstrated that only specific groups of immune cells responded to AG intervention and immunocompromised cells were more likely regulated by AG treatment.

## 1. Background

American ginseng (*Panax quinquefolius *L., AG) is one of the major tonics used in traditional Chinese medicine for the prevention and treatment of consumptive respiratory infection and other diseases for about 300 years [1]. Together with Asian ginseng, AG is now among the top 5 of the commonly used herbal medicines for improving physical and psychological performance as well as immunomodulation in the United states [2]. Many animal and human cell studies demonstrate a wide range of pharmacological effects of American ginseng on the regulation of both innate and adaptive immunity [3–7]. However, conflicting results can also be found. For example, in an *ex vivo* study, a polysaccharide-rich extract of American ginseng named COLD-fX (CX) was found to be able to increase Con-A-induced spleen IL-2 and IFN-*γ* (Ifng) productions from C57BL/6 (B6) mice in a dose-dependent manner [8]. On the contrary, in an *in vivo* study, the same product was found to decrease spleen IL-2 and IFNG production in Sprague-Dawley rats following Con-A and/or LPS stimulation for 24 or 48 h [9]. This variation cannot be attributed to the frequently mentioned reason, that is, different ginsenoside content and composition [6, 10]. Since the tumor incidence varies greatly in normal Sprague-Dawley rats [11] and B6 mice (http://jaxmice.jax.org/strain/000664.html), we assumed that the different pathophysiological states may have major impacts on the distinct responses to American ginseng treatment.

In this study, we investigated this assumption by performing a comparative microarray assay to determine the immunomodulatory effect of American ginseng in murine splenic cells of different pathophysiological states *ex vivo*. Ginsenosides and polysaccharides are two major bioactive components of American ginseng involved in the modulation of the immune system [12]. But a recent study shows that polysaccharides may mediate most of the immunomodulatory properties of American ginseng. So the polysaccharide-rich American ginseng product CX was used for this study. However, individual bioactive ingredients can hardly explain the emergent properties of plant systems [13, 14]. Therefore, a crude powder of American ginseng root was chosen to make aqueous extract of American ginseng equivalent to tea-like preparation of American ginseng in Chinese medicine practice. The results showed that American ginseng exhibited exclusively strong immunomodulatory activities in immunocompromised murine immune cells. Specifically, the Ifng pathway was found to be significantly suppressed by American ginseng in this group of cells.

## 2. Methods

### 2.1. American Ginseng

The crude powder of American ginseng (CP) was purchased from Sigma-Aldrich (St. Louis, MO, USA). This product has been used as American ginseng standard in research community [15, 16]. The dried powder were homogenized in sterilized phosphate-buffered saline (PBS, pH 7.4) and heated at 90°C for 20 minutes, followed by filtering through a 0.45 *μ*M Millipore membrane to remove particulate material and any bacterial contaminants, and used fresh the same day. Since the bioactive ingredients of American ginseng are relatively small molecular weight products, the homogenization and filtering should not have affected their activity or concentration in the final preparation. To ensure the repeatability and consistency for each test, the same lot of the product was used to prepare the working solution by the same person (YM).

COLD-fX (CX) was purchased from the Natural Vitamin Direct (Burnaby B.C. Canada). According to the manufacturer (CV Technologies, Edmonton, AB, Canada), COLD-fX is composed of 80% poly-furanosyl-pyranosyl-saccharides, 10% protein, and 10% mixture of residual moisture, trace amounts of amino acids, vitamins, minerals, and small organic molecules. In contrast to most of other American ginseng products, the extract contains no ginsenosides. This product has been intensively studied recently as immunomodulator [9, 17]. The powder content of COLD-fX capsule was dissolved in phosphate-buffered saline (PBS, pH 7.4), filtered through 0.45 *μ*m Millipore membranes to remove particulate material and any bacterial contaminants, and used fresh the same day.

### 2.2. Cells

C57BL/6 mice were purchased from the Jackson Laboratory (Bar Harbor, ME, USA). Mice of seven weeks old and gender mixed were used for the study. All animal care and experiments were performed under institutional protocols approved by the Institutional Animal Care and Use Committee at the University of Tennessee Health Science Center (UTHSC) and Veterans Administration Medical Center (VAMC) at Memphis. 

Splenocytes were prepared by disrupting the spleen with a syringe in complete medium (RPMI 1640 with 10% fetal bovine serum, 1% penicillin-streptomycin, and 10 mM HEPES). After a 10 min centrifugation at 300 ×g to separate debris, the cells were washed in RPMI medium, followed by lysis of erythrocytes using ammonium chloride reagent (BD Biosciences, San Jose, CA, USA). The cells were then counted and viability was determined by trypan blue exclusion. Splenic cells were resuspended at appropriate densities for use in subsequent assays.

### 2.3. Preparation of Working Cells of Different Functional States

To prepare cells for the study, the cells obtained above were divided into 4 groups to mimic 4 basic pathophysiological states: (1) normal naïve; (2) normal activated; (3) deficient naïve; (4) deficient activated. They were cultured with saline, concanavalin A (ConA, Sigma Cat. no. C5275, 1 *μ*g/mL), dexamethasone (DEX, from Sigma Cat. no. D4902, 1 *μ*g/mL for 2 h) plus saline, and DEX plus Con A, respectively.

### 2.4. Flow Cytometric Analysis

Cultured splenocytes (1 × 10^6^/sample) were stained with fluorochrome labelled anti-mouse antibodies specific for CD3 and CD25 surface markers (BD Biosciences) for 30 minutes at 4°C. Labelled cells were washed with PBS, and a minimum of 100,000 cells was analysed for each sample with BD LSR II flow cytometer (BD Biosciences). The final analysis was performed by using FlowJo software (Tree Star, Ashland, OR, USA).

### 2.5. American Ginseng Treatment

1 × 10^5^ above prepared cells were suspended in triplicate into wells of 96-well U-bottom microtiter plates followed by serial 10-fold increasing concentrations of the American ginseng extracts from 0.01 *μ*g/mL to 1000 *μ*g/mL or medium alone [18]. Optimal dose was determined based on their effects on cell proliferation measured using MTT method below.

### 2.6. MTT Assay of Splenic Cell Proliferation

Splenocytes were suspended in RPMI-1640 containing 10% fetal bovine serum. 100 *μ*L cells were seeded into the 96-well plates (Coastar, Corning, NY, USA) at a density of 3 × 10^5^/well. Three wells were included in each group. After 24 h incubation, MTT test was performed using CellTiter 96 Nonradioactive Cell Proliferation Assay Kit (Promega, Madison, WI, USA) according to the manufactory protocol. Briefly, 15 *μ*L of the Dye Solution was added to each well of the plate for 4 h incubation. Then 100 *μ*L of the solubilization solution/stop mix was added to each well and incubated overnight in a sealed container with a humidified atmosphere. The absorbance at 570 nm (OD reading) was quantified using a 96-well plate reader (DYNATECH MR 4000). Cell proliferation rate (CPR) was calculated using formula: CPR = [(OD_exp_ – OD_con_)/OD_con_] × 100%, where OD_exp_ is the value of optical density of experimental group and OD_con_ is the value of optical density of control group.

### 2.7. Microarray Assay

In each group, triplicate cell samples were collected from three repeat cultures. Total RNAs were isolated from these cells using Trizol Reagent (Life Technologies, Carlsbad, CA, USA) according to the manufacturer's instructions. The RNAs were purified by RNeasy MinElute Cleanup Kit (Qiagen, Valencia, CA, USA) and quantified using NanoDrop-2000 (Thermo, Wilmington, DE, USA). The integrity of the RNAs was evaluated by Bioanalyzer 2100 (Agilent, Santa Clara, CA, USA). Samples with a RIN (RNA integrity score) of more than 8 were used for cDNA synthesis with the Illumina TotalPrep RNA Amplification Kit (Life Technologies). Labelled cDNA samples were hybridized overnight to the Mouse-6 v2.0 BeadChip in a multiple step procedure according to the manufacturer's instructions. The chips were washed, dried, and scanned on the BeadArray Reader (Illumina, San Diego, CA, USA) and raw data were generated using GenomeStudio 3.1 (Illumina) and normalized using quantile normalization algorithm.

### 2.8. Real-Time PCR

For each sample, 50 ng of total RNA was used for TaqMan Real-time PCR with the probes from Life Technologies (Table 1). The PCR reactions were carried out with an ABI 7900 Real-Time PCR System using ABI's standard protocol. Relative gene expression change was calculated with ddCt method using GAPDH as internal control.

### 2.9. Statistical Analysis

For microarray data analysis, Partek Genomics Suite software (Partek, St. Louis, MO, USA) was used to generate differentially expressed gene lists with one-way ANOVA. Genes with a fold change of ≥2 and a *P* value with false discovery rate (FDR) of <0.01 were selected. Functional annotation clustering was done using the Functional Annotation tool of the DAVID Bioinformatics Resources 6.7 [19]. To identify the Ifng regulated genes among the differentially expressed genes, Interferome software was used to profile the effect of American ginseng on the Ifng signaling functions [20].

For other assays, results were expressed as the mean ± standard deviation (S.D.) of three experiments and were compared using paired-samples *t*-test with IBM SPSS Statistics 21 (IBM, Armonk, NY, USA). Differences were significant at *P* < 0.05.

## 3. Results

### 3.1. Characterisation of Different Functional Groups of Murine Splenic Cells

**Figure 1:**
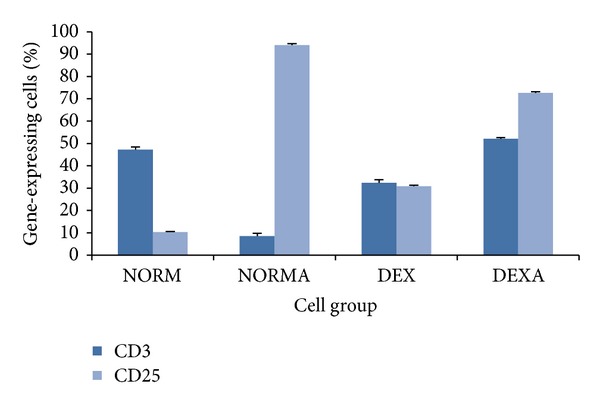
Flow cytometry analysis of different groups of splenic cells. Murine cells were divided into four groups, which were treated with saline, ConA, DEX, and DEX/ConA, respectively, as described in Section 2. CD25 as T lymphocyte activation marker was upregulated in both NORMA and DEXA groups, whereas the expression of CD3 as T lymphocyte surface marker changed in dual direction possibly due to different activation status in these two groups. NORM = normal naïve; NORMA = normal activated; DEX = deficient naïve; DEXA = deficient activated.

**Figure 2:**
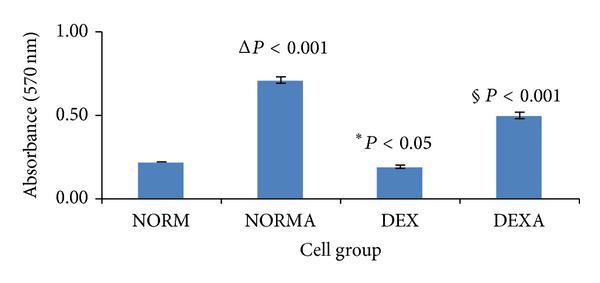
Proliferation status of different groups of splenic cells by MTT assay. Murine cells were divided into four groups, which were treated with saline, ConA, DEX, and DEX/ConA, respectively, as described in Section 2. After 24 h incubation, MTT assays were performed to evaluate the proliferation status of the cells. Triplicates were averaged to generate the final results. Compared with the naïve groups (NORM and DEX), the cells of activated groups (NORMA and DEXA) showed higher proliferation rate. In addition, the cells of DEX group showed lower proliferation rate compared with the NORM group. Δ denotes comparison between normal naïve (NORM) and normal activated (NORMA) groups; § denotes comparison between deficient naïve (DEX) and deficient activated (DEXA) groups; ∗ denotes comparison between normal naïve (NORM) and deficient naïve (DEX) groups.

To determine if the pathophysiological state has any possible impact on the immune modulatory effect of American ginseng, the separated murine splenic cells were first treated differently to mimic 4 basic pathophysiological states: (1) normal naïve; (2) normal activated; (3) deficient naïve; (4) deficient activated. ConA is able to stimulate T cells [21] and DEX is a potent immunosuppressive agent, capable of directly affecting the function of lymphocytes [22].  Figure 1 shows the percentage of T lymphocytes expressing CD3 and CD25 in each group, validating the success of the cell modeling.  Figure 2 shows distinct proliferation profiles of different groups of cultured murine splenic cells before American ginseng treatment. The OD of deficient naïve group was significantly lower than normal naïve group (*P* < 0.05), whereas the ODs were significantly higher in the activated groups compared with the naïve groups (*P* < 0.001), indicating the inhibition and promotion of cell proliferation by DEX and ConA, respectively.

### 3.2. Effect of American Ginseng on the Proliferation of Splenic Cells

**Figure 3:**
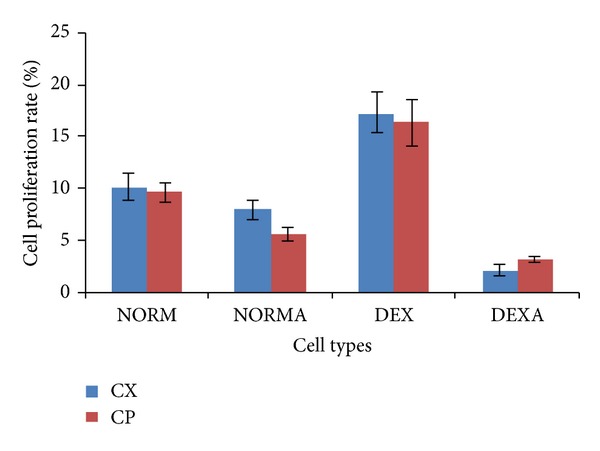
Effects of American ginseng on proliferation of murine splenic cells *ex vivo*. After 24 h incubation with American ginseng, the cells were collected for MTT assays to determine the effect of American ginseng on the proliferation of murine splenic cells in different groups. Compared with the cell proliferation of saline-treated control subgroups, cells in American ginseng treated subgroups of NORM and DEX groups exhibited higher rate of cellular proliferation. But no significant difference was observed between CP and CX subgroups (*P* > 0.05). CX = COLD-fX; CP = crude powder of American ginseng root.

Before investigating possible differential molecular mechanisms underlying the immunomodulating effects of CP and CX in different groups of cells, proliferation assays were performed to determine the phenotypic changes associated with the American ginseng treatment in terms of cell proliferation rate (CPR). Different concentrations of ConA stimulation and American ginseng treatment were tested and the best combination of ConA (1 *μ*g/mL) and American ginseng (62.5 *μ*g/mL) for maximum cell proliferation effect was determined for further microarray assays.  Figure 3 showed that cell proliferation was increased significantly in the naïve groups compared with the active groups (*P* < 0.001).

### 3.3. Distinct Immunomodulating Signatures Induced by American Ginseng in Different Groups of Murine Splenic Cells

**Figure 4:**
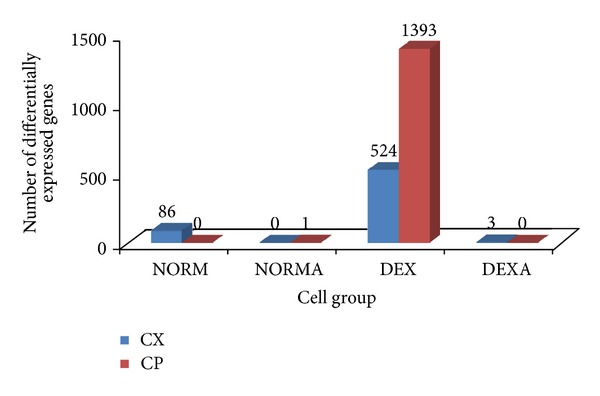
Effects of American ginseng on global gene expression in different groups of murine splenic cells. Cells of different functional status were treated with two different types of American ginseng products, that is, CX (COLD-fX) and CP (crude powder). After 24 h incubation, the cells were collected for total RNA isolation. Microarray assays were performed on individual samples (three each group) and data were analyzed using Partek Genomics Suite software. Differentially expressed genes were identified in four different types of cells. The numbers above each bar represent the number of differentially expressed genes.

To determine the influence of cell physiological state on the change of global gene expression profiles in response to American ginseng, microarray assays were performed to identify differentially expressed genes between different groups. The results showed that both CX and CP induced big gene expression changes in the DEX group (Figure 4). Some changes of gene expression were also induced by CX, not CP, in the NORM group See Supplementary Material (S1) available online on http://dx.doi.org/10.1155/2013/972814. But the expressions of much more genes were affected by CP than CX in the DEX group (S2 and S3). Among those differentially expressed genes induced by American ginseng in the DEX group, 161 and 149 were found to be downregulated and upregulated in common by both CX and CP, respectively.  Table 2 shows the expression changes of common known genes induced by the two American ginseng products in the DEX group.

### 3.4. Gene Functional Clusters Regulated by American Ginseng Treatment

To identify specific gene functional clusters regulated by American ginseng in different groups, DAVID Bioinformatics Resources 6.7 was used to analyze the three gene sets (S1–S3). With the S1 gene set, a cluster of gene associated with membrane-enclosed lumen was identified from the upregulated genes, whereas many functional clusters of genes were identified with the other two data sets (Table 3).

### 3.5. Downregulation of Ifng Signal Pathway Induced by American Ginseng in the DEX Group of Murine Splenic Cells

A recent microarray study of other similar American ginseng extracts shows that Ifng is the most significantly upregulated gene in healthy human immune cells [4]. However, to our surprise, the present study showed downregulation of Ifng expression exclusively in the DEX group (Table 1). To find more evidence of the downregulation, Interferome software was used to profile the effect of American ginseng on the expression of Ifng responsive genes (Table 4).

### 3.6. Validation of Ifng Signaling Pathway Downregulation Using Real-Time PCR

To further validate one of our new findings, real-time PCR assays were performed to determine the expression of several major downregulated genes associated with Ifng signaling, including Cxcl10, Gbp1, Gbp2, Ifng, Indo, Irf1, Jun, Stat1, Stat2, and Tbx21.  Table 5 shows the distinct expression pattern of these genes in different groups of cells. Most of changes identified by our microarray assay were confirmed by the real-time PCR assay with variable extent, indicating possible sensitivity variation of the two methods.

## 4. Discussion

Interindividual response variation is widespread in the application of herbal medicine, such as American ginseng. Systems pharmacology may provide a new angle for better understanding of the complicated drug-response phenotypes [23]. In this study, we used animal cell models to explore the possible mechanisms for this phenomenon with gene expression microarray technology and found that distinct physiologic state-associated molecular mechanisms may explain the variations of murine spleen cells in response to American ginseng treatment.

Our comparative microarray data showed that a great deal of gene expression changes was induced in the immune deficient group of cells, suggesting that this type of cells may be major targets of American ginseng treatment. In addition, different mechanisms by which American ginseng worked were identified in different functional groups. For example, membrane-enclosed lumen involved genes was upregulated in the normal naïve group treated with CX, while in the deficient naïve group treated with CX, one of the major changes was the upregulation of signal peptide-encoding genes expression (Table 2). Moreover, great variations of gene expression changes were also identified between groups where two American ginseng products (CX and CP) were used. In the NORM group, the expression of some genes was regulated by CX, but not by CP (Figure 4). But in the DEX group, although 208 known genes were regulated by both products (Table 1), many more genes were regulated by CP than CX (Figure 4). 

Ifng is a cytokine critical for innate and adaptive immunity against viral and intracellular bacterial infections and for tumor control [24]. But its activities and regulation may be dependent on the cellular, microenvironmental, and/or molecular context [25], which may partially explain the dual effects of American ginseng on the Ifng expression (Table 4) and the conflicting reports. Tbx21 is a potent transactivator of the Ifng gene and functions as the master regulator of Th1 lineage commitment [26]. Jun is also involved in the regulation of Ifng expression together with Stat4 in TCR-triggered T cells [27]. So the downregulation of Tbx21 and Jun may be mainly accountable for the decreased Ifng expression in the DEX group (Table 4). Cxcl10, Gbp1, Gbp2, Indo, Irf1, and Stat1 represent the major components of the Ifng signaling pathway and main Ifng responsive genes [28]. The decreased expression of these genes in the DEX group revealed the evidence for the suppression of the Ifng signaling activities and functions. 

A recent study shows that low Ifng production due to a single nucleotide polymorphism at the first intron of Ifng gene significantly increases the possibility to achieve extended longevity in a group of Italian centenarians [29]. Ageing is characterized by a Ifng driven chronic, low grade, Th-1 type inflammation which could contribute to the onset of major age-related psychiatric conditions (such as depression, anxiety, insomnia, and cognitive impairment) and medical diseases (such as cardiovascular diseases, neurodegeneration, osteoarthritis and osteoporosis, and diabetes) [30, 31]. There is now emerging evidence that Ifng may also be involved in the development of aggressive tumors [25]. In this regard, inhibition of the Ifng pathway through American ginseng may be a viable new approach to healthy ageing and longevity in some immune compromised populations.

American ginseng was originally applied in the treatment of pulmonary infection as lung tonic [32]. The finding of the increased expression of lysozyme genes (Lyz2, Lyz, Lyzs), Ltf, and Slpi with CX and CP exposure (Table 1, S2, and S3) in the DEX group may provide a good reason for this application. Rapid elimination of inhaled microorganisms from the airways and distal lung airspaces is essential for pulmonary host defense. Antimicrobial proteins/peptides play a key role in promoting a sterile gas exchange surface by directly killing and/or facilitating phagocytosis of microorganisms by resident lung macrophages. Recent studies show that the majority of bactericidal activity in the respiratory passages appears to be contributed by lysozyme, lactoferrin (LTF), and secretory leukoprotease inhibitor (SLPI) [33, 34]. In this study, all of these genes were highly regulated by American ginseng in the DEX group. Especially, Lyz2 was identified to be the most upregulated known gene in response to both CX and CP treatment (Table 2, S2, and S3). 

## 5. Conclusions

In summary, the present comprehensive microarray study demonstrates that the functional status may have major impacts on the response to American ginseng treatment in murine immune cells. This finding may provide supporting scientific evidence for personalized application of American ginseng in the prevention and treatment of disease.

Most of the studies on the standardization and characterization of medical plants focus on the analysis of a limited number of “marker” compounds. Frequently, however, the overall activities of medicinal plants are not well understood, and, therefore, their analysis should not be biased towards a few abundant or easily detected compounds. From a system's biological point of view, multiple components of a herb can act through additive or synergistic mechanisms to impart a greater biologic effect than can be achieved by any component in isolation. This statement is validated by the great variations of CP and CX on the gene expression change in this study. 

Given the importance of the Ifng pathway in the development of many chronic diseases and longevity, the finding of the downregulation of the Ifng pathway induced by CP and CX in the DEX group may help pave a novel approach to improving public health. 

## Supplementary Material

The Supplementary Materials file consists of three tables: *Table NORM-CX* lists the differentially expressed genes regulated by COLD-fX (CX) in the normal naïve group of murine splenic cells; Table DEX-CX lists the differentially expressed genes regulated by CX in the deficient naïve group of murine splenic cells; *Table DEX-CP* lists the differentially expressed genes regulated by Crude Powder of American Ginseng (CP) in the deficient naïve group of murine splenic cells.

## Figures and Tables

**Table 1:** Probe list for real-time PCR.

Gene	Probe_ID
Cxcl10	Mm00445235_m1
Gbp1	Mm00657086_m1
Gbp2	Mm00494575_m1
Ifng	Mm00801778_m1
Indo	Mm00492586_m1
Irf1	Mm01288580_m1
Jun	Mm00495062_s1
Stat1	Mm00439531_m1
Tbx21	Mm00450960_m1

**Table 2:** Common gene expression changes induced by two different types of American ginseng products in murine splenic cells *ex vivo*.

Gene symbol	Fold-change induced by CX	Fold-change induced by CP	Gene symbol	Fold-change induced by CX	Fold-change induced by CP
Aicda	−2.9	−3.1	Ruvbl2	−2.1	−3.2
Ankrd37	−3.2	−2.8	Scd2	−3.3	−3.2
Apitd1	−2.1	−2.3	Sco1	−2.7	−4.0
Asf1b	−2.1	−3.4	Serpina3f	−24.3	−12.5
Atf3	−4.1	−3.1	Serpina3g	−10.2	−5.8
Auh	−2.2	−2.1	Sgol2	−2.3	−3.4
Aurkb	−2.4	−4.8	Siah2	−2.3	−3.1
Birc5	−2.2	−5.4	St6galnac4	−2.1	−2.2
Brip1	−2.2	−2.7	Stat1	−3.0	−2.7
Brrn1	−2.3	−3.9	Tbx21	−6.7	−5.6
Car13	−2.1	−2.4	Tcf19	−2.3	−3.2
Ccdc99	−2.3	−2.5	Tg	−2.7	−3.8
Ccnd2	−2.4	−2.4	Tmem97	−2.0	−3.0
Ccne1	−3.0	−2.9	Top2a	−2.0	−3.4
Ccr5	−3.1	−3.1	Tpi1	−2.9	−3.7
Cd86	−2.8	−2.1	Tpx2	−2.1	−3.7
Cdca2	−2.6	−2.9	Tuba3b	−2.3	−2.3
Cdca3	−2.5	−4.1	Upp1	−2.4	−2.3
Cdca5	−2.9	−3.2	Wars	−2.9	−2.7
Cdkn3	−2.2	−2.5	Zbtb32	−5.3	−8.1
Cenpi	−2.1	−2.8	Actn1	2.2	2.1
Cenpn	−2.7	−3.2	AI467606	2.2	2.7
Cep55	−2.2	−3.8	Alox5ap	2.1	2.6
Chaf1a	−2.3	−3.4	Anxa3	2.2	3.9
Chaf1b	−2.0	−2.7	Apoe	3.1	4.0
Chchd6	−2.1	−2.7	Apol7c	4.8	4.4
Clspn	−2.1	−4.1	Arhgef18	2.1	2.9
Cox6a2	−3.1	−10.6	Arsj	2.9	4.7
Cxcl10	−6.6	−5.9	Atg16l2	2.0	2.5
Dlgap5	−2.0	−2.7	Bcl11b	2.1	2.2
E2f1	−2.1	−2.9	Ccl9	2.2	3.2
Esco2	−2.0	−3.9	Cd27	2.1	2.1
Espl1	−2.2	−2.2	Cd68	2.3	2.3
Fancd2	−2.5	−3.1	Cd8b1	2.0	2.5
Fanci	−2.3	−2.4	Clec4d	2.6	2.5
Fcgr4	−4.2	−2.8	Clec4n	2.3	3.0
Fdps	−2.1	−2.8	Crhbp	4.1	5.0
Ffar2	−16.3	−13.5	Crxos1	2.0	2.5
Fgl2	−4.9	−4.5	Cxcl4	2.5	3.4
Galk1	−2.0	−2.8	Egr2	3.1	2.1
Gbp1	−2.0	−2.0	Egr3	3.1	2.7
Gbp2	−7.6	−4.9	Emb	2.1	2.1
Gbp3	−3.6	−2.4	Emr1	3.5	2.6
Gbp6	−3.8	−3.0	Ephx1	2.2	4.5
Gins1	−2.7	−4.2	Etsrp71	2.8	3.8
Gmnn	−2.0	−2.1	Faim3	2.9	3.4
Gmppb	−2.6	−2.5	Fcer2a	2.0	2.6
Gng12	−3.1	−3.4	Flrt3	2.9	3.8
Gpr109a	−2.3	−2.1	Gad1	2.0	3.1
Grhpr	−2.7	−2.2	Gli3	2.2	2.7
Hist1h2ag	−2.3	−2.7	Gpnmb	3.0	4.4
Hist1h3c	−2.4	−2.2	H2-M2	4.0	3.3
Hist2h2ab	−2.5	−2.7	Icam2	2.0	2.6
Hnrpab	−2.1	−2.6	Il11ra1	2.1	2.6
Hyou1	−2.0	−2.4	Il7r	2.2	2.5
Ifi47	−4.5	−3.4	Irx5	2.4	3.2
Ifng	−11.1	−7.3	Kcnrg	2.5	2.4
Iigp2	−3.1	−2.5	Kctd11	2.0	2.0
Il12rb1	−2.5	−2.4	Klrd1	2.6	3.9
Incenp	−2.2	−2.7	Kpnb3	3.3	3.2
Indo	−2.6	−2.3	Lgals3	2.1	4.2
Ipo5	−2.0	−2.9	Lpl	2.9	4.3
Irg1	−5.8	−5.6	Ltf	3.3	6.6
Irgb10	−6.7	−6.7	Ly116	2.3	3.4
Isg20	−3.7	−3.0	Lyz	4.2	5.0
Jun	−3.7	−3.2	Lyz2	7.3	6.7
Kif11	−2.5	−3.7	Lyzs	3.8	4.9
Kif15	−2.6	−2.9	Mafg	2.2	2.3
Kif22	−2.1	−2.8	Mdga2	2.7	2.3
Kif4	−2.7	−3.2	Mmp9	2.1	2.7
Kntc1	−2.1	−3.4	Msmb	2.4	2.0
Lgals9	−2.2	−2.3	Nagk	2.2	3.2
Lig1	−2.4	−3.0	Ngp	3.1	2.8
Ly6a	−2.4	−2.2	Nrp1	2.1	2.2
Mad2l2	−2.4	−2.1	Pcdhga2	2.2	2.6
Mcm10	−2.2	−5.2	Pdcd4	2.0	2.7
Mcm2	−2.2	−2.4	Pira3	2.2	2.7
Mcm7	−2.1	−3.1	Prl4a1	2.3	2.3
Mid1ip1	−2.5	−2.9	Punc	2.4	2.7
Mlkl	−3.3	−4.4	Rab5b	2.3	2.7
Mrps28	−2.2	−2.2	Rasl2-9	2.3	2.0
Mybl2	−2.7	−2.8	Rgl2	2.1	2.8
Ncapd2	−2.2	−2.6	Rgs10	2.4	2.8
Ncaph	−2.2	−4.5	Rnf122	2.1	3.0
Ndc80	−2.3	−2.0	Rnu6	2.3	2.6
Ndufb9	−2.1	−2.1	S100a8	3.4	4.0
Nmral1	−3.9	−3.6	S100a9	2.9	4.7
Noc4l	−2.4	−2.4	Sesn1	2.1	3.2
Nudt1	−2.2	−2.1	Sgk1	2.1	2.8
Nusap1	−2.2	−2.8	Siat7c	2.6	3.4
Oasl1	−7.7	−3.8	Sirpa	2.2	3.7
Oosp1	−2.0	−3.2	Slc11a1	3.1	2.5
Paics	−2.0	−2.3	Slc40a1	2.7	3.4
Pdss1	−2.1	−3.5	Smpdl3a	2.0	3.4
Pgk1	−2.1	−3.6	Snn	2.0	2.2
Phf11	−6.9	−3.4	Sspn	2.2	4.0
Phf19	−2.6	−2.6	Tax1bp3	2.1	2.3
Pkm2	−2.0	−2.5	Tmem71	2.1	2.0
Plk1	−2.2	−4.3	Tmie	2.4	2.8
Pole	−2.1	−3.0	Trat1	2.2	2.9
Prc1	−2.0	−3.6	Trp53inp1	2.0	2.0
Psmb9	−3.8	−2.3	Vmn2r42	3.1	2.8
Rrm1	−2.6	−3.4	Wdr9	2.0	2.3
Rrm2	−2.4	−3.2	Wnt10a	3.8	2.1

**Table 3:** Gene functional clusters regulated by American ginseng in murine splenic cells *ex vivo*.

Group	Change	Term	Genes
NORM-CX	Upregulation	Membrane-enclosed lumen	POLR2F, PNO1, CHCHD4, MRTO4, ATF5, CDCA8, TIMM8A1, C1QBP, MRPL17, SDF2L1, RANGRF, GEMIN6, TFDP1

DEX-CX	Upregulation	Signal peptide	MPZL3, NRP1, MSMB, MMP9, CRHBP, CCL9, ARSJ, GREM1, IL7R, CD68, SMPDL3A, APOE, LTF, EMB, GPNMB, CD27, PRL2C2, SHBG, WNT10A, LPL, LYZ2, CD8B1, ICAM2, TMIE, MDGA2, IL11RA1, STIM1, SIRPA, ACPL2, TMEM66, EMR1, PRL4A1, SLPI, FAIM3
	Downregulation	Cell cycle	E2F1, CLSPN, CCDC99, PRC1, KNTC1, AURKB, CEP55, CCNE1, NCAPH, MCM7, FANCI, INCENP, CDCA2, CDCA5, CDCA3, KIF11, DLGAP5, SGOL2, LIG1, GMNN, NUSAP1, BIRC5, NDC80, MCM2, ESCO2, ATM, NCAPD2, FANCD2, CCND2, PLK1, SIAH2, CHAF1A, MAD2L2, CHAF1B
		DNA metabolic process	GINS1, KIF22, CLSPN, NUDT1, LIG1, POLE, TREX1, BRIP1, MCM2, MCM10, ESCO2, ATM, CCNE1, MCM7, FANCI, FANCD2, RRM2, RRM1, AICDA, RUVBL2, CHAF1A, TOP2A, CHAF1B
		Cellular response to stress	KIF22, CLSPN, NUDT1, LIG1, POLE, BRIP1, TREX1, ESCO2, ATM, FANCD2, FANCI, IFNG, RUVBL2, EIF2AK2, CHAF1A, CHAF1B
		Nucleotide binding	KIF22, TUBA3B, OAS2, AURKB, GMPPB, WARS, GALK1, PTK2, MCM7, IGTP, NT5C3, OASL2, KIF4, TAP1, OASL1, GBP10, MLKL, MX2, TOP2A, DHX58, GBP6, KIF11, GIMAP7, BC006779, LIG1, POLE, KIF15, IFI47, BRIP1, TREX1, GRHPR, MCM2, ATM, ABCG1, PSMB9, HYOU1, GVIN1, PLK1, PKM2, RRM1, RUVBL2, PGK1, EIF2AK2, OAS1G, GBP3, PAICS, GBP2, GBP1
		Intracellular nonmembrane-bounded organelle	GYPC, KIF22, CLSPN, ZBTB32, CCDC99, PRC1, HIST1H2AG, TUBA3B, KNTC1, CEP55, AURKB, DAXX, HIST2H2AB, PTK2, FANCI, INCENP, KIF4, ASF1B, TOP2A, DBNL, CENPN, KIF11, MRPS28, NOC4L, DLGAP5, SGOL2, KIF15, TPX2, NUSAP1, BIRC5, NDC80, CSRP1, MCM2, MID1IP1, ATM, CENPI, NCAPD2, APITD1, FANCD2, HIST1H3C
		DNA binding	E2F1, KIF22, CLSPN, HIST1H2AG, TBX21, MYBL2, HIST2H2AB, MCM7, LOC100046232, KIF4, LOC100048299, TOP2A, DHX58, LIG1, POLE, NUSAP1, BRIP1, TREX1, MCM2, STAT1, ESCO2, ATM, STAT2, TRIM30, ATF3, APITD1, JUN, IRF7, IRF8, IRF1, HIST1H3C, RUVBL2

DEX-CP	Upregulation	Cytoplasmic membrane-bounded vesicle	SELP, RAB5B, CAMP, HEXB, RASL2-9, TGFB3, ACTN1, VEZT, CHI3L3, ANXA2, RABAC1, ATP7A, SLC11A1, SYN2, SORT1, LTF, MPO, NEU1, GPNMB, SLC40A1, RIN3, RAB27A
		Signal	NRP1, PLXNA2, FAM20B, CRHBP, MMP9, HEXB, SORL1, ARSJ, PGLYRP1, TGFB3, RETNLG, SIDT1, CD1D1, CD97, TMEM108, LOC100046259, SERPINE2, SLC24A3, APOE, SMPDL3A, LTF, IZUMO1, SEPP1, GPC1, DPP7, RAMP1, NXPH1, RAMP3, WNT10A, CD3G, CD3D, CRTAC1, ICAM2, CAR11, CAMP, TMIE, MDGA2, LRP1B, PTPRR, CST3, IL11RA1, H2-DMB1, SIRPA, HCST, CD84, CCDC3, ACVR2B, H2-OA, PRL4A1, LOC100047936, BACE1, SORT1, FAIM3, ERN2, FCRLA, NEU1, PRNP, CASQ2, CPM, IGFBPL1, MSMB, ENPP2, CLM3, CCL9, FCGRT, CCL5, IL7R, CD68, ITGB7, SFTPD, FCER1G, EMB, GPNMB, CD27, TYROBP, LPL, SELP, OVGP1, KLK8, LYZ2, CD8B1, PTPRZ1, PSAP, NID1, CHI3L3, HGF, IL6RA, CD55, EMR1, CXCL16, LIPH, MPO, LYG1
		Regulation of apoptosis	LST1, NUAK2, MMP9, STK17B, TGFB3, GLI3, GPX1, TSC22D3, NOD1, APOE, BCL11B, TRP53INP1, FCER1G, LOC100047353, LTB, PIK3R1, RASA1, CD27, RAB27A, CD3G, HGF, SNAI2, ATP7A, NRP, ADRB2, MSX1, ERN2, PRNP
	Downregulation	Cell cycle	E2F1, RAD51C, CLSPN, PRC1, KNTC1, AURKA, AURKB, CCNE1, CDCA8, MCM7, SEH1L, OIP5, FANCI, INCENP, PSMC3IP, MTBP, CDCA2, RANBP1, H2AFX, TUBG1, CDCA5, ASPM, CDCA3, CDC6, KIF11, DSN1, SGOL2, LIG1, SGOL1, TPX2, MND1, NUSAP1, ESPL1, MCM2, MCM3, CDK4, ESCO2, 6720463M24RIK, RAD51, MCM6, NCAPD2, UHRF1, MAD2L1, TIMELESS, SPAG5, FANCD2, CCND2, BUB1B, LOC640972, SIAH2, STMN1, MAD2L2, NUP43, CCDC99, TIPIN, ANLN, CEP55, RCC1, C79407, SPC25, NCAPH, NCAPG2, F630043A04RIK, MNS1, TFDP1, CKAP2, MKI67, DLGAP5, GMNN, NASP, SYCE2, BIRC5, CDC20, NDC80, CDKN3, CENPH, CCNB1, PLK1, PHGDH, CHTF18, CHAF1A, CHAF1B
		Intracellular nonmembrane-bounded organelle	RPP38, PRC1, KNTC1, AURKA, AURKB, EBNA1BP2, TOP1, CDCA8, OIP5, INCENP, PRIM2, H2AFX, TUBG1, RPS27A, ASPM, NUP133, SGOL2, SGOL1, RRP9, MRTO4, NCAPD2, RSL1D1, PA2G4, MAD2L1, RFC4, SPAG5, STMN1, MYBBP1A, NUP43, HMGB2, CCDC99, BLM, LMNB1, NHP2L1, NOC3L, TIPIN, ANLN, BANF1, SPC25, ORC6L, HIST1H4F, MNS1, ASF1B, MRPS27, CKAP2, MRPS28, NOC4L, MKI67, MRPS22, SYCE2, NDC80, PLK4, NUP62, HIST1H3A, PCNA, HIST1H3C, DNMT1, HIST1H3D, HIST1H3E, TMPO, ZBTB32, CLSPN, KIF22, LYAR, MKI67IP, GTSE1, SLC1A4, KIF2C, HIST2H2AB, RRP1B, GRWD1, SEH1L, FANCI, RANBP1, TOP2A, FTSJ3, CDC6, KIF11, EXOSC6, DSN1, KIF15, EXOSC2, TPX2, NUSAP1, NUP85, MRPS6, MCM2, MID1IP1, POLR1B, LOC100047827, RAD51, APITD1, FANCD2, NOL10, BUB1B, NUP107, KPNA2, WDR43, 2610036L11RIK, MTDH, HIST1H2AG, TUBA3B, UTP6, DNAHC11, CEP55, FCF1, C79407, GPHN, KIF4, MRPL16, F630043A04RIK, MARS, CENPN, TCP1, CENPM, RRP12, DLGAP5, PNO1, CENPP, BIRC5, CENPK, CENPI, CENPH, CCNB1, HIST1H2AH, HIST1H2AK, MPHOSPH6
		Chromosome	ZBTB32, CLSPN, KIF22, KNTC1, AURKB, HIST2H2AB, TOP1, CDCA8, OIP5, SEH1L, FANCI, INCENP, PRIM2, H2AFX, TUBG1, TOP2A, NUP133, DSN1, SGOL2, SGOL1, NUP85, MCM2, NCAPD2, LOC100047827, RAD51, MAD2L1, RFC4, APITD1, SPAG5, FANCD2, BUB1B, NUP107, NUP43, 2610036L11RIK, HMGB2, CCDC99, BLM, HIST1H2AG, TIPIN, BANF1, C79407, SPC25, F630043A04RIK, ORC6L, HIST1H4F, ASF1B, CENPN, CENPM, TCP1, MKI67, CENPP, SYCE2, NDC80, BIRC5, CENPK, CENPI, CENPH, HIST1H3A, PCNA, HIST1H2AH, DNMT1, HIST1H3C, HIST1H2AK, HIST1H3D, TMPO, HIST1H3E
		DNA metabolic process	CLSPN, KIF22, RAD51C, MCM10, CCNE1, TOP1, MCM7, FANCI, PRIM2, PSMC3IP, H2AFX, TOP2A, CDC6, NUDT1, POLH, LIG1, POLE, GTF2H4, MND1, MCM2, RBBP7, MCM3, ESCO2, MCM5, MCM6, RAD51, UHRF1, RFC4, FANCD2, RRM2, RRM1, LOC640972, AICDA, RUVBL2, HMGB2, BLM, UNG, TIPIN, BANF1, TK1, ORC6L, APEX1, FEN1, GINS1, GINS2, RAD51AP1, NASP, BRIP1, EEF1E1, POLD2, PCNA, DNMT1, CHTF18, CHAF1A, CHAF1B
		ATP binding	HSP90AB1, RAD51C, KIF22, NARS, FIGNL1, CTPS, AURKA, CAD, CCT3, AURKB, MTHFD1, DDX27, WARS, TOP1, KIF2C, MCM7, OASL1, MLKL, TOP2A, CDC6, KIF11, HSP90AA1, PFKL, LIG1, AARS, KIF15, PFKP, TBRG4, CCT6A, PBK, MCM2, MCM3, CDK4, GMPS, MCM5, TTF2, RAD51, MCM6, TARS, NME2, RFC4, PKM2, RARS, EIF4A1, RRM1, FARSB, BUB1B, RUVBL2, ALDH18A1, BLM, TRIB3, ASNS, DNAHC11, KARS, PFAS, TK1, IARS, GALK1, STK40, KIF4, LARS, HSPE1, UCK2, HSPA5, HSPA8, MARS, TCP1, PIF1, PDK3, DDX1, BRIP1, EPRS, AARSD1, LOC100046163, PSMB9, GART, HYOU1, CCT5, PLK4, PLK1, CHTF18, HSPD1, PGK1, PAICS
		Nucleotide binding	RAD51C, CTPS, HMGCR, AURKA, AURKB, CCT3, DDX27, TOP1, OASL1, MLKL, TUBG1, GBP6, LIG1, AARS, POLE, TBRG4, IFI47, NME2, RFC4, RARS, SNRPA, GBP3, GBP2, GBP1, BLM, KARS, TK1, GMPPB, EIF3B, GFM1, HSPE1, PIF1, DDX1, BRIP1, EPRS, AARSD1, VDAC2, VDAC3, LOC100046163, GART, PSMB9, HYOU1, CCT5, PLK4, PLK1, HSPD1, HSP90AB1, KIF22, NARS, FIGNL1, MKI67IP, CAD, MTHFD1, WARS, KIF2C, MCM7, SRPR, TOP2A, CDC6, KIF11, HSP90AA1, PFKL, KIF15, PFKP, CCT6A, GRHPR, PBK, MCM2, CDK4, ARL6, MCM3, GMPS, MCM5, TTF2, RAD51, MCM6, TARS, SQLE, PKM2, EIF4A1, RRM1, FARSB, BUB1B, THOC4, RUVBL2, ALDH18A1, TUBA3B, TRIB3, ASNS, DNAHC11, PFAS, IARS, GALK1, GPHN, STK40, KIF4, LARS, HSPA5, UCK2, GAPDH, HSPA8, MARS, TCP1, PDK3, PHGDH, CHTF18, PGK1, PAICS
		Membrane-enclosed lumen	E2F1, RPP38, PDIA3, LYAR, EZH2, PDIA6, MKI67IP, TOP1, EBNA1BP2, CDCA8, RRP1B, OIP5, GRWD1, TOP2A, FTSJ3, EXOSC6, GTF2H4, EXOSC2, NUSAP1, RRP9, POLR1B, CDK4, RBBP7, MRTO4, RSL1D1, PA2G4, TIMM8A1, C1QBP, JUN, NOL10, THOC4, RUVBL2, WDR43, MYBBP1A, MDH2, ALDOA, MTDH, LMNB1, NHP2L1, UTP6, NOC3L, TIMM10, CHCHD4, CALR, FCF1, KDELC1, SET, MRPL16, CACYBP, HSPE1, HSPA5, WDHD1, GEMIN6, TFDP1, MARS, RRP12, NOC4L, MKI67, MRPS22, PDK3, PNO1, ATF5, HYOU1, PLK4, ATF3, SDF2L1, PCNA, HSPD1, MPHOSPH6
		Cellular response to stress	KIF22, CLSPN, HMGB2, BLM, UNG, TIPIN, MIF, FANCI, IFNG, BCL3, H2AFX, HSPA5, FAM129A, APEX1, FEN1, RAD51AP1, NUDT1, POLH, LIG1, AARS, POLE, GTF2H4, BRIP1, ESCO2, RAD51, UHRF1, NUPR1, TIMELESS, FANCD2, EEF1E1, PCNA, RUVBL2, LOC100044948, CHAF1A, CHAF1B

**Table 4:** List of Ifng regulated genes suppressed by American ginseng (CP and CX) in the DEX group of murine splenic cells.

Gene	Description
Atf3	Activating transcription factor 3
Cxcl10	Chemokine (C-X-C motif) ligand 10
Fdps	Farnesyl diphosphate synthetase
Gbp1	Guanylate nucleotide binding protein 1
Gbp2	Guanylate nucleotide binding protein 2
Gbp3	Guanylate nucleotide binding protein 3
Hyou1	Hypoxia upregulated 1
Ifi47	Interferon gamma inducible protein 47
Ifng	Interferon gamma
Indo	Indoleamine-pyrrole 2,3 dioxygenase
Isg20	Interferon-stimulated protein
Jun	Jun oncogene
Kif11	Kinesin family member 11
Lgals9	Lectin, galactose binding, soluble 9
Mybl2	Myeloblastosis oncogene-like 2
Pgk1	Phosphoglycerate kinase 1
Psmb9	Proteasome (prosome, macropain) subunit, beta type 9 (large multifunctional peptidase 2)
Rrm1	Ribonucleotide reductase M1
Stat1	Signal transducer and activator of transcription 1
Tmem97	Transmembrane protein 97
Top2a	Topoisomerase (DNA) II alpha
Upp1	Uridine phosphorylase 1
Wars	Tryptophanyl-tRNA synthetase

**Table 5:** Verification of downregulation of Ifng pathway in the DEX group of murine splenic cells.

Gene	NORM (fold change)		NORMA (fold change)		DEX (fold change)		DEXA (fold change)	
	CX	CP	CX	CP	CX	CP	CX	CP
Cxcl10	−4.2	−3.8	2.2	1	−39	−9	2	3.5
Gbp1	1.8	1.3	−1.1	−1.8	−6.7	−4.6	1.6	2.8
Gbp2	1	−1.75	1.2	1	−17.5	−3.9	1.3	1.7
Ifng	1	1	1.9	1.7	−54.6	−25.9	−3.4	−29
Indo	−3.3	−1.7	2.5	2.2	−3	−3.1	3.6	8.5
Irf1	−1.1	−1.2	−1.2	−1.4	−3.4	−1.4	1.2	2.1
Jun	−1.2	−1.5	1.1	1	−1.2	1	1	2.4
Stat1	−1.2	−1.1	1	−1.2	−5	−1.4	1	1.5
Tbx21	−4.7	−4.9	1.1	1	−21	−7.6	1.7	2

## Abbreviations

AG: American ginseng
CX: COLD-fX
CP: Crude powder of American ginseng
ConA: Concanavalin A
Dex: Dexamethasone
Ifng: Interferon-*γ*.
